# New York Odontological Society

**Published:** 1885-06

**Authors:** 


### NEW YORK ODONTOLOGICAL SOCIETY.

The April meeting of this society was held at the residence of Dr. J. W. Clowes, 667 Fifth Ave., Dr. W. Jarvie, Jr., presiding.

Dr. Clowes read a paper on "Separations," in which he made allusion to a recent visit from a gentleman who had been his patient for thirty-five years, and who thanked the doctor for having filed his teeth apart many years ago, feeling convinced that but for the separations then made, his teeth would not have been so well preserved. Approximal decay had found no place to lurk, and plenty of room had been afforded to keep all the surfaces clean. Dr. Clowes referred to a paper on the same subject, which he read before the Odontological Society on a previous occasion, and which provoked sharp, adverse criticism, and an emphatic protest against such practice on the part of one gentleman in particular, who was considered an authority in his profession. This gentleman had spoken of the annoyances experienced from such separations, and the difficulty in mastication which it caused. He also stated that he had been called upon to restore, by contour fillings, teeth to cut away. "His harangue," said Dr. Clowes, "seemed to have a marked effect on his hearers, who possibly felt that to cut away teeth would greatly wrong their patients." A venerable friend, however, who happened to be present on that occasion, defended Dr. Clowes' practice, and alluded to the fact that the South Sea Islanders were in the habit of filing the approximal surfaces of their teeth, and with no apparent injury.

Dr. Clowes had practiced his method of separation for many years, and had never as yet received a complaint from any one so treated, yet he felt sure that his patients would not hesitate to make complaints if there was occasion for it. The doctor referred to "the approximal arch and lingual bevel" as a sure safeguard against approximal decay.

Dr. J. Smith Dodge, Jr., read an interesting paper on the Relation of Nerve Forces to the Teeth.

Dr. J. Morgan Howe considered Dr. Dodge's paper one of the ablest papers ever read before the society. He fully believed with the writer that nerve force had much to do with the supply of material to the teeth, but he did not believe the lime salts to be alive, as he understood the essayist to state in his paper.

*Prof. Frank Abbott*—Said that the paper expressed his own views on the subject. He had often advised patients to take more out of door exercise, and thereby help their digestion, for he believed this a cause of much dental trouble.

*Dr. W. H. Atkinson*—Was glad to have lived long enough to hear such a paper read before a dental society, and thought several evenings should be spent in discussing it. He wished there might be a full expression of the meeting concerning it.

*Dr. N. W. Kingsley*—Said that while listening to the paper, he envied the writer very much, and at the same time he had derived much pleasure in hearing the views of the essayist so beautifully expressed. He believed that the nervous system had much to do with the decay of teeth.

*Dr. E. Parmly Brown*—Thought salt was one of the factors of decay.

